# Prospects for lithium-ion batteries and beyond—a 2030 vision

**DOI:** 10.1038/s41467-020-19991-4

**Published:** 2020-12-08

**Authors:** Clare P. Grey, David S. Hall

**Affiliations:** 1 Department of Chemistry, University of Cambridge, Lensfield Road, Cambridge, CB2 1EW UK; 2 Faraday Institution, Becquerel Ave, Harwell Campus, Didcot, OX11 0RA UK

**Keywords:** Batteries, Batteries

## Abstract

It would be unwise to assume ‘conventional’ lithium-ion batteries are approaching the end of their era and so we discuss current strategies to improve the current and next generation systems, where a holistic approach will be needed to unlock higher energy density while also maintaining lifetime and safety. We end by briefly reviewing areas where fundamental science advances will be needed to enable revolutionary new battery systems.

Lithium-ion batteries (LIBs), while first commercially developed for portable electronics are now ubiquitous in daily life, in increasingly diverse applications including electric cars, power tools, medical devices, smart watches, drones, satellites, and utility-scale storage. As battery usage multiplies, so do the specific requirements, with increasing divergence of battery designs and sizes to suit each specific use. A pressing challenge—especially over the next decade—is to develop batteries that will make a significant contribution to reducing and eventually eliminating carbon emissions, in some countries including the UK as early as 2050, to mitigate global warming. Current LIBs are fit for frequency regulation, short-term storage and micro-grid applications, but expense and down the line, mineral resource issues, still prevent their widespread on the grid. There are many alternatives with no clear winners or favoured paths towards the ultimate goal of developing a battery for widespread use on the grid.

Present-day LIBs are highly optimised, operating for months-to-years, with some expected to function for decades. This is a considerable achievement, given that many of the materials operate outside their thermodynamic stability windows. The anodes (negative electrodes) are lithiated to potentials close to Li metal (~0.08 V vs Li/Li^+^) on charging, where no electrolytes are stable. Instead, the battery survives by forming a passivation layer, or solid-electrolyte interphase (SEI), preventing further electrolyte degradation. On the cathode side, Al current collector corrosion is mitigated by the decomposition of the electrolyte salts, again, producing a stable passivation layer. Cathode materials have been optimised to minimise oxygen loss at higher temperatures to help prevent ‘thermal runaway’, and to withstand the mechanical stresses of the repeated volume changes associated with Li removal and insertion.

While some advances were serendipitous, most were the result of extensive and global research efforts, leading to a highly optimised system fit for many purposes. Consequently, our current commercial systems contain materials that are operating with energy densities operating increasingly closer to their fundamental limits, i.e., further lithium removal from the cathode results in irreversible structural transformations or oxygen loss, while on the anode no vacancies in the lattice remain to accommodate more Li ions. The separators and current collectors are becoming thinner, and batteries are being pushed to higher voltages via surface coatings, electrolyte additives, and morphology optimisation.

It would be unwise to assume ‘conventional’ LIBs are approaching the end of their era; many engineering and chemistry approaches are still available to improve their performance. While much research focusses on making improvements to single components, a holistic approach will be needed to unlock higher energy density while also maintaining lifetime and safety.

Resources are also critical with massive increases in production. The move away from LiCoO_2_ (LCO) (in portables) to Ni-rich materials in EVs (addressing Co mining concerns), means that Ni resources become critical too. This has motivated a re-evaluation of the use of the lower voltage cathode material LiFePO_4_. The question then becomes, where next? The route from a lab-scale development to market is long, and since this comment focusses on a 2030 vision, we highlight research likely to impact our world in the current decade, but then touch briefly on work needed to achieve global zero-carbon (ZC) goals in the coming decades.

## Optimisation of current commercial and related chemistries

This is an area with massive ongoing global fundamental and applied research effort. A strong focus is on mitigating degradation, to increase longevity (and indirectly cost), and because degradation becomes more severe as the voltages are increased, and, for example, more Ni and Si are added to the cathode and anode, respectively. It is also hoped that learning from these studies can be generalised and applied to the next generation of battery chemistries. These studies are aided by the impressive development of new experimental and theoretical tools and methodologies, including operando measurements that can study batteries that are closer to the practical device, with improved temporal and spatial resolution and increased sensitivity. In the case of NMR spectroscopy, one area that the authors focus on, dynamic nuclear polarisation (DNP) methods, involving the transfer of magnetisation from unpaired electrons to nuclear spins, has been used to enhance the signal of the SEI, or more recently to examine the Li metal–SEI interface^1^. Moving forward, the DNP method is likely to play an increasingly important role in examining the buried interfaces ubiquitous in batteries. We now discuss some specific challenges in more detail.

### Cathodes

**Fig. 1:**
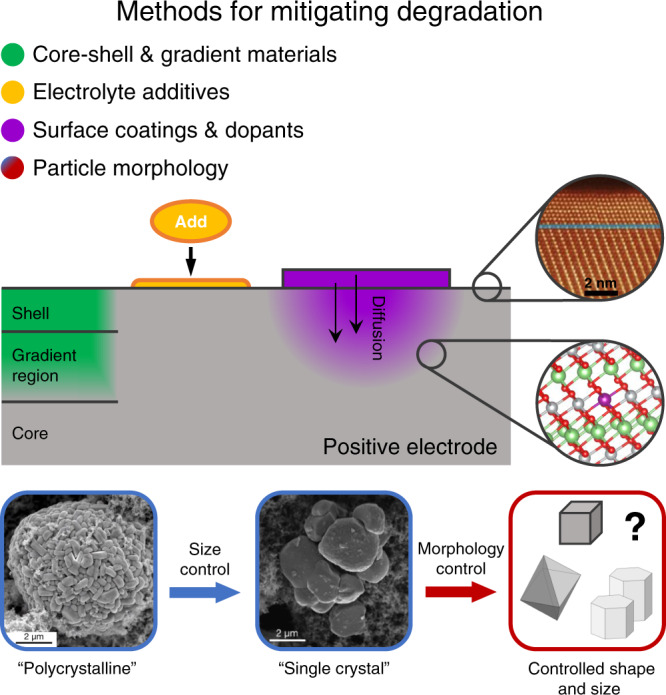
Potential approaches for improving lifetime of LiMO_2_ positive electrodes. Core-shell and gradient materials utilise more stable compositions (often lower Ni-content) near the electrode surface to minimise electrode-electrolyte reactivity and a nickel-rich core stoichiometry to increase energy density. Electrolyte additives are compounds added to the electrolyte solution on the order of a few weight per cent to improve cell lifetime and safety, for example by reacting with the electrode surface to form a protective ‘barrier’ layer. Surface coatings (applied via a variety of methods) on the electrode material can improve cycling stability and lifetime by scavenging corrosive HF, physical blockage of electrolyte components from reaching the electrode surface, slowing RSL growth by blocking oxygen loss from the active material, and via other chemical reactions with the electrolyte components. Heat treatments of surface-coated materials can be used to prepare surface-doped materials with improved chemical stability and that inhibit the growth of surface rock-salt layers. One trend in particle morphology research is to increase primary particle sizes (i.e., transition from polycrystalline to ‘single crystal’ materials), while future prospects include the synthesis of finely tuned particle shapes and sizes. (TEM of RSL adapted from Lin et al.^14^).

Figure 1 summarises current and future strategies to increase cell lifetime in batteries involving high-nickel layered cathode materials. As these positive electrode materials are pushed to ever-higher voltages and nickel contents, increased rates of electrolyte oxidation and surface rock-salt layer (RSL) growth become increasingly problematic for maintaining practical cell lifetimes, RSL formation generally leading to impedance rise^2,3^. RSL formation and the concomitant loss of oxygen have been proposed to be the primary driver of electrolyte oxidation at high voltages, rather than Faradaic currents—affecting materials from LiNiO_2_ through to LCO^4,5^. Yet many fundamental questions remain. What chemical factors determine the rate of oxygen diffusion and RSL growth? Why (and when) is singlet oxygen observed and how does it form? Are electrolyte components oxidised at the electrode surface or in the solution? Higher nickel content is also associated with larger anisotropic volume changes during cycling—representing a source of intra- and inter-granular cracking—and ‘fatigued’ phases with lower practical capacity.

While the timeline to establish answers is uncertain, these and other basic questions will almost certainly be increasingly studied and debated in the coming years. New understanding will allow for more strategic development of methods to mitigate degradation pathways (Fig. 1). Core-shell particles could be prepared with optimised gradients of different transitional metal and s/p-block metals, and layer thicknesses with stable surfaces and higher energy density cores—following on from a number of pioneering studies^6^; surface coating stoichiometries and doping elements could be chosen to lower the rate of oxygen loss and RSL formation; surface-modifying electrolyte additives could be designed to inhibit singlet oxygen evolution and to slow electrolyte oxidation. The development of detailed micromechanical models will guide particle morphology optimisation—size and shape—for various materials and applications. However, all of these possible advances hinge on the ability of the field to connect fundamental concepts with the complex multi-process behaviour of modern LIBs and ultimately to demonstrate that this leads to longer lifetime. For this, increased fundamental understanding, obtained via careful experimental and theoretical studies, is required.

### Anodes

An ‘obvious’ win involves replacing graphite with either silicon or silicon oxide, due to their fivefold–tenfold higher energy densities. However, this is not straightforward: SiO_x_ causes considerable first cycle irreversibly capacity loss associated with the formation of inorganics such as Li_2_O and Li_4_SiO_4_^7^. A stable SEI does not form on silicon, in part because of the large volume expansion that is a direct consequence of its large capacity. While its first cycle irreversible capacity loss is lower, it is currently difficult to achieve high enough coulombic efficiencies for applications needing >300–500 cycles. Many current commercial cells include small amounts of SiO_x_ (2–10%) into graphite anodes, providing modest capacity gains. Polymer and graphene (carbon) coatings (and mesostructures/shells) coupled with different electrolyte additives have all been proposed to increase coulombic efficiencies and enable the use of higher Si contents. Alternatively, limiting the range over which the silicon is lithiated minimises the volume expansion, leading to a more stable SEI. Graphite–Si composites bring with them other challenges including the mechanical grinding of graphite caused by the Si expansion/contraction. Calendaring graphite to increase its practical volumetric energy density will result in more mechanical grinding. While Si will play a role in future battery technologies, a question remains as to the extent and the degree to which the longevity of cells and safety will win out over increased energy density. The answers will vary across sectors, Si mostly likely playing a larger role in batteries where lifetime and safety are less critical.

### Electrolytes and other cell components

To increase the volume fraction occupied by active electrode materials—again reducing cost—current collectors and polymer separators have become much thinner over the years. Higher loadings can also be achieved by increasing the active layer thicknesses, decreasing the binder fraction, and decreasing the porosity. All of these require increased electrolyte (ionic) transport to maintain rate capability, an area of active research already for fast-charging battery technologies^8^. The transport properties and molecular-scale structures of new solution chemistries (e.g., new solvent systems, highly concentrated salts) are becoming increasingly understood^9,10^. Basic studies—both experiments and calculations—of the physicochemical properties of new electrolyte compositions are expected to continue leading to new materials and insight into their properties. Beyond this, the structure and stability of the SEI in various solutions and conditions (temperature, voltage) must be better characterised. Such insights will feed development of optimised additive/coatings for enabling alternative electrolytes, while maintaining cell lifetimes. Intensive benchmarking and lifetime analysis of these systems remains a present and future need. Finally, their cost and safety of handling will need to be proven before wide or large-scale adoption is possible, the latter representing an important but underrepresented area of study.

## Next generation materials and batteries

Here strategies can be roughly categorised as follows:

1. The search for novel LIB electrode materials.
2. ‘Bespoke’ batteries for a wider range of applications.
3. Moving away from traditional liquid electrolytes—e.g., ionic liquids, high salt content electrolytes, and solid state batteries (SSBs).
4. Enabling anion redox chemistries—Li air, Li-sulphur and beyond.
5. Moving beyond Li: Na, Mg, Ca, Al.
6. Decoupling electrochemistry and storage—redox flow batteries.

The search for novel LIB electrode materials is an area with considerable challenges. While new materials or morphologies are reported with regularity, to be commercially relevant, they must be scalable. Volumetric and gravimetric energy densities must reflect those of an electrode and not just of those of the materials itself, i.e., rate performance must be demonstrated for an electrode that contains sufficient active material to provide the required energy density for the application in question. Relatively early on, the Materials Project mined all of the inorganic structure data base (ICSD) and materials proposed via data mining algorithms (including simple swaps of elements while keeping the structure type fixed)—at that time more than 10,0000 materials. While, considerable insight was obtained into what structural features control voltage etc. only a limited number of new classes of battery materials were discovered. For example, carbonophosphates were identified, which represented a mineral structure type that had not previously synthesised and tested in battery applications^11^. Subsequent structure prediction activities have generated many (meta)stable structures, but the challenge remains to identify structures that are stable on cycling, for example to oxygen loss particularly at the top of charge, or more generally, to structural reorganisations. Even if a structure is predicted, it is not currently easy to predict if and how they can be synthesised^12^.

An area that has received considerable recent attention is coupled transition metal–anion redox. While established in sulphur-based chemistry, where sulfide ions, S^2−^, can be readily and reversibly oxidised to persulfides, S_2_^2−^, and to elemental sulphur (in lithium–sulphur batteries), there are distinct differences when the anion is an oxide ion. The higher O^2−^/O^−^ redox couple means anion redox can occur simultaneously with cation redox chemistry providing higher capacities and coupled processes. Challenges are associated with the often-accompanying instability towards oxygen loss and structural changes that accompany Li removal. The latter can result in hysteresis between charge and discharge and the ‘voltage droop’ seen in so-called Li excess materials. While not directly linked, many of these chemistries are associated with poor rates,. However, the ‘Li excess materials’ contain higher Mn contents than typical EV-type cathode materials, and so have the potential to be both cheaper and more environmentally friendly further motivating their study. The next 10 years will see increased understanding as to how these materials function and how oxygen loss can be mitigated. Perhaps applications will emerge where they can make an impact?

We have not touched on the wide range of electrode materials, explored now over many years, which involve displacement or conversion chemistries, where lithiation (or sodiation) results in partial-to-complete rearrangement of lattices. Here challenges include rate performance, voltage hysteresis, and lifetime. Lithium metal continues to attract considerable attention as an anode, but Li dendrite formation remains a concern, providing considerable incentive to push towards all solid-state batteries (SSBs) with solid state electrolytes.

None of the beyond Li chemistries are straightforward, with the possible exception of Na, where many of the learnings for LIBs can be applied. But even here, there are distinct differences, due to the larger size of Na which favours different coordination environments and lattices (e.g., graphite cannot accommodate Na), and the higher solubility of the Na salts in the SEI, which means that different electrolyte additives are required.

One question that is worth reflecting on is the degree to which new emerging—or small more ‘niche’ markets can tolerate new battery chemistries, or whether the cost reductions associated with scale will always favour usage of a limited set of battery chemistries. Lithium titanium oxide (LTO) currently has a relatively modest market in applications—including fast charging—where safety and the ability to operate over a wide temperature window are issues: the anode material operates at 1.55 V vs. Li, where neither Li plating nor conventional SEI formation are an issue. Alternatives to LTO are being developed which include niobium titanium oxide (NTO) by Toshiba and niobium tungsten oxide compounds in our laboratory, with potential applications in small-to-grid scale batteries. Batteries with different voltages may be more suitable for new microelectronics applications (e.g., as the voltage demands for computer chips drop), removing the need for DC-DC conversion, and being more readily coupled with energy harvesting electronics. Small primary batteries are currently used to power some remote sensors. These are projected to be needed in their billions-to-trillions to power internet of things (IoT) devices, requiring a considerable workforce to replace them, often from difficult locations^13^. Could new rechargeable batteries be produced at a low enough cost for the different often bespoke applications? Medical batteries can tolerate higher price margins perhaps allowing batteries with different materials to be developed, but here reliability and safety will be paramount.

It is the belief of the authors that fundamental science will be key to overcoming the many and diverse fundamental roadblocks in the ‘beyond LIB’ space To make step changes in battery performance, we must increasingly learn how to control metastable materials—from their initial synthesis, to their stability in non-equilibrium and harsh environments—be it temperature or voltage. We must learn how to control interfacial structures—from the SEI, to the interfaces between two components in a solid state-state battery. Better structural models of these interfaces are needed, to improve our ability to compute the relevant processes with realistic computational resources, and improve our understanding of how they function. Ideas of self-healing systems have emerged in the polymer space and have been suggested as potential safety shut-down mechanisms, but looking forward, these concepts must translated into cathode and anode chemistry. We must continue to develop new methods to increase our understanding of the multiple non-equilibrium processes in batteries: with increasing technology demands, coupled with ZC goals that dictate reduced and more sustainable energy usage, the need for basic and applied research is more important than ever, with many fundamental scientific challenges remaining in the road ahead.
